# Differential type I and type III interferon expression profiles in rheumatoid and juvenile idiopathic arthritis

**DOI:** 10.3389/fmed.2024.1466397

**Published:** 2024-09-27

**Authors:** Anikó E. Malik, Drew Slauenwhite, Sarah M. McAlpine, John G. Hanly, Jean S. Marshall, Beáta Dérfalvi, Thomas B. Issekutz

**Affiliations:** ^1^IWK Health Centre, Halifax, NS, Canada; ^2^Department of Pediatrics, Faculty of Medicine, Dalhousie University, Halifax, NS, Canada; ^3^Division of Rheumatology, Faculty of Medicine, Dalhousie University, Halifax, NS, Canada; ^4^Queen Elizabeth II Health Sciences Centre, Halifax, NS, Canada; ^5^Department of Microbiology & Immunology, Faculty of Medicine, Dalhousie University, Halifax, NS, Canada

**Keywords:** rheumatoid arthritis, juvenile idiopathic arthritis, interferon, dendritic cell, synovial fluid, cytokine

## Abstract

**Background:**

The role of type I and type III interferons (IFNs) in rheumatoid arthritis (RA) and juvenile idiopathic arthritis (JIA) is still poorly understood. The objective of this study was to examine the hypothesis that IFN expression profiles in the peripheral blood differ between subsets of arthritic subjects. Multiple type I and type III IFNs were examined in patients with RA and JIA, as well as among subtypes of JIA.

**Methods:**

Treatment-naïve RA and JIA patients were enrolled. Droplet digital PCR was used to measure the expression of type I, II, and III interferons in blood and synovial fluid leukocytes. Dendritic cell subsets were isolated from synovial fluid to examine IFN expression in each subset. Additionally, synovial mononuclear cells and JIA-derived fibroblast-like synoviocytes were stimulated with TNF, IFNγ, and poly(I:C) to examine inducible IFN expression.

**Results:**

The predominant type I IFN gene expressed by blood leukocytes was *IFNκ* and was significantly lower in RA than JIA and controls. Oligoarticular and psoriatic JIA subgroups showed higher *IFNκ* expression compared to polyarticular JIA and RA. JIA synovial fluid leukocytes expressed abundant *IFNγ* and type III IFNs (*IFNλ1, IFNλ3*), with distinct dendritic cell subset contributions. JIA fibroblast-like synoviocytes produced IFNβ, IFNλ1, and IFNλ2 mRNA upon poly(I:C) stimulation.

**Conclusion:**

This study revealed differences in IFN expression patterns in RA and JIA, with notable differences between JIA subtypes. The expression levels of *IFNκ, IFNγ*, *IFNλ1 and IFNλ3* in JIA suggest specific roles in disease pathology, influenced by disease subtype and joint microenvironment. This study contributes to understanding IFN-mediated mechanisms in arthritis, potentially guiding targeted therapeutic strategies.

## Introduction

Rheumatoid arthritis (RA) is a complex systemic disease characterized by heterogeneous clinical presentations, ranging from mild symptoms to severe inflammation and early joint destruction. This clinical heterogeneity reflects diversity within cellular and molecular immunopathology (1). Similarly, juvenile idiopathic arthritis (JIA) is the most common chronic rheumatologic disease of childhood, with multiple subtypes including oligoarticular, polyarticular, and psoriatic JIA (2). In both RA and JIA, prognosis and treatment responses vary widely, highlighting the need for biological markers to guide treatment choices.

The latest approved class of disease-modifying antirheumatic drugs (DMARDs), Janus kinase (JAK) inhibitors, offers promising therapeutic options for patients refractory to treatment with conventional DMARDs and biologics (3). The JAK/signal transducer and activator of transcription (STAT) pathways are crucial in interferon (IFN) signaling, underscoring the importance of understanding IFN contributions to autoimmune arthritis.

IFNs have been implicated in the pathogenesis of RA and JIA (4). IFNs are pleiotropic cytokines divided into three groups termed type I (α1-13, *β*, *ε*, *κ* and ω1), type II (IFNγ), and type III (λ1, λ2, λ3, and λ4) (5, 6). Although type I and type III IFNs are structurally distinct, they have overlapping functions, and while binding to specific receptors they both signal through the JAK/STAT pathway to induce transcription of IFN-stimulated genes (ISGs) (7). This study focuses on type I and type III IFNs, with type II IFN examined for comparison, since its role in arthritis has been thoroughly investigated (8).

Current knowledge indicates that type I IFNs (IFNα and IFNβ) play significant roles in RA and JIA pathogenesis, contributing to inflammation, joint destruction and autoimmunity (9). Type I IFNs activate various cell types crucial in RA pathogenesis, including macrophages, dendritic cells (DC), B cells, and fibroblast-like synoviocytes (FLS), enhancing the production of inflammatory cytokines like IL-6, matrix metalloproteinases, and autoantibodies. Clinical trials blocking type I IFN signaling pathways using monoclonal antibodies against their receptor subunit IFNAR1 have shown efficacy in reducing RA disease activity (10). However, there is still much to learn about the expression patterns of other potent type I IFNs such as IFNε, IFNκ, and IFNω1, which also signal through the type I IFN receptor and have not been widely studied in these conditions. By studying a broader range of type I IFNs, we can potentially identify new therapeutic targets and gain a better understanding of their roles in disease pathology.

Type III IFNs exert both pro- and anti-inflammatory effects on innate and adaptive immunity (11). IFNλ1 enhances the production of pro-inflammatory cytokines and chemokines, leading to immune cell recruitment to sites of inflammation (12). IFNλ2 also has anti-inflammatory effects including inhibition of neutrophil reactive-oxygen-species production, degranulation, formation of neutrophil-extracellular-traps, and chemotaxis (13, 14). This anti-inflammatory effect is similar to IL-10, with which IFNλs share sequence homology and signaling through the shared IL-10R2 subunit (15).

In RA, serum IFNλ1 has been shown to be elevated, particularly among autoantibody-positive patients, but no correlation was observed with disease activity (DA) (16). There is no published data on the expression and function of type III IFNs in JIA.

While IFNα and IFNβ are known to be expressed in the blood and synovium of RA and JIA patients (17, 18), the expression of other type I and type III IFNs in blood leukocytes and inflamed synovial fluid cells has not been studied. Moreover, FLS in the synovial lining mediate joint destruction by perpetuating inflammation via cytokine production (including type I IFNs) (19). Type I IFN expression in JIA has also not been examined.

We hypothesize that there are distinct differences in the expression of type I and type III IFNs between RA and JIA and among subtypes of JIA, which could be significant for disease mechanisms, potential response to treatment, and outcomes. We observed a previously unrecognized dysregulation of IFNκ and notable differences between JIA and RA profiles as well as altered expression among subsets of JIA patients. This study contributes to our understanding of IFN-mediated mechanisms in arthritis. By identifying IFN expression patterns in RA and JIA, as well as among different JIA subtypes, this study seeks to provide insights into disease pathology and guide the development of targeted anti-IFN therapies. This could lead to more effective therapeutic strategies for patients with RA and JIA.

## Materials and methods

### Patients and sample selection

Treatment-naïve RA patients who met the 2010 American College of Rheumatology (ACR)/European League Against Rheumatism (EULAR) classification criteria were recruited from the Rheumatology Clinic at The Arthritis Center, Queen Elizabeth II Health Sciences Center in Halifax, Canada (20). Patients with moderate-to-high disease activity [Disease Activity Score in 28 joints using the erythrocyte sedimentation rate (DAS28-ESR of >3.2)] and no previous exposure to glucocorticosteroids were included. Clinical parameters including disease duration, C-reactive protein (CRP), erythrocyte-sedimentation rate (ESR), and autoantibody serostatus [of rheumatoid factor (RF) and anti-citrullinated protein antibodies (ACPA) were obtained]. Response to therapy was determined based on DAS28-ESR score of ≤3.2 after 6 months of methotrexate (MTX) treatment. Treatment-naïve oligoarticular, polyarticular, and psoriatic JIA patients without exposure to glucocorticosteroids and fulfilling the International League Against Rheumatism (ILAR) classification criteria for JIA were enrolled from the Rheumatology Clinic at IWK Health in Halifax, Canada (21). Exclusion criteria for all groups included individuals with current infections, malignant diseases, and other systemic autoimmune diseases. Healthy volunteers of similar age and sex to the RA patients with no infections or systemic autoimmune diseases were included as healthy controls (HCs). Studies were approved by the Nova Scotia Health Authority and IWK Health research ethics boards. All participants or legal guardians provided written informed consent.

### Blood and synovial fluid sample processing

Heparinized blood was collected from all patients. Platelet-free plasma was separated, erythrocytes were lysed, leukocytes were washed twice with phosphate-buffered saline (PBS), then lysed in RNA lysis buffer (Qiagen) and stored at −80°C.

Synovial fluid (SF) was collected from inflamed knee joints of treatment-naïve JIA patients in acid citrate dextrose. Cell-free SF was separated, bulk SFL were washed twice in PBS, lysed in Qiazol (Qiagen), and stored at −80°C. SF mononuclear cells (SFMNCs) were purified by layering over Ficoll-Paque Plus, and centrifuged at 540 x g for 25 min. SFMNCs were washed three times using RPMI-1640 containing 10% fetal bovine serum (Gibco) and stored in liquid nitrogen.

### Sorting of dendritic cell subsets

SFMNCs of three oligoarticular JIA patients were stained with the antibodies panel in Supplementary Table 1, washed, resuspended in PBS containing albumin, and sorted using BD FACS Aria Fusion (BD Biosciences).

### Establishment and activation of fibroblast-like synoviocytes

Primary FLS were cultivated from SF samples of treatment-naïve JIA patients, as previously described (22). Activation assays were initiated on the fourth passage at 80% confluency. Each culture was treated with TNF, IFNγ (PeproTech), or the TLR3 ligand high molecular weight poly(I:C). After 6 h, supernatants were removed, and cell monolayers were lysed with 700 μL Qiazol.

### Total RNA extraction and cDNA synthesis

Total RNA was extracted from 600 μL blood leukocyte (BL) and synovial fluid leukocyte (SFL) lysates using RNeasy Mini Kit (Qiagen) as per the manufacturer’s instructions. Total RNA extracts were stored at −80°C. Synthesis of cDNA from 200 ng total RNA per reaction was performed using an iScript™ cDNA Synthesis Kit (Bio-Rad) and cDNA samples were stored at −20°C.

### Reverse Transcription Quantitative Real-Time PCR

ISG expression (ISG15, OAS1, IFIT1, CXCL10) in leukocytes and IFN expression in FLS were assessed in duplicate by qPCR using SYBR Green (Bio-Rad) on the CFX Connect Real-Time Thermocycler (Bio-Rad) for a total of 40 cycles. *Actin Gamma 1 (ACTG1)* and *Hypoxanthine Phosphoribosyl Transferase 1* (*HPRT1*) served as reference genes. Negative controls (‘no reverse transcriptase’ and no template controls) were included according to MIQE (Minimum Information for Publication of Quantitative Real-Time PCR Experiments) guidelines (23).

### Droplet digital PCR

IFN expression (IFNλ1, IFNλ2, IFNλ3, IFNα1, IFNβ1, IFNε, IFNκ and IFNω1) in leukocytes was assessed by ddPCR using EvaGreen Supermix (Bio-Rad) and primers described in Supplementary Table 2. Droplets were generated using a QX200 Droplet Generator and thermocycling was performed in a C1000 Touch Thermocycler (Bio-Rad). Droplets were read on a QX200 Droplet Reader (Bio-Rad). The absolute quantity of DNA per sample (copies/μL) was calculated using QuantaSoft (v.1.7.4.0917). The relative expression was calculated by ∆C_T_ method.

### ELISA

IFNλ1 protein level in diluted (1:4) plasma and synovial fluid duplicate samples from JIA patients was measured using enzyme linked immunosorbent assay (ThermoFisher) as per the manufacturer’s instruction.

### Statistical analyses

Data are presented as individual points with medians. Statistical significance was determined using the non-parametric Mann–Whitney or Wilcoxon Sign Rank test. Linear correlation coefficients were calculated using Spearman’s correlation test. Statistical analyses were performed using GraphPad Prism (GraphPad Software). *p* values <0.05 were considered statistically significant.

## Results

### Characteristics of study subjects

The RA cohort included 35 DMARD-naïve patients with recent-onset RA. After 6 months of MTX treatment, 15 patients (42.9%) achieved remission based on their DAS28-ESR score, while 20 patients (57.1%) had persistent moderate-to-high disease activity. No significant differences were observed in mean age, gender distribution, baseline DAS28-ESR score, CRP level, ESR, or ACPA/RF serostatus between the two groups (Supplementary Table 3). The JIA cohort included 16 treatment-naïve patients (61% female) of oligoarticular (56%), polyarticular (25%), and psoriatic (19%) subtypes, enrolled at the time of intraarticular steroid injection.

### *IFNκ* was the major type I IFN expressed in the blood leukocytes of both RA and JIA patients

**Figure 1:**
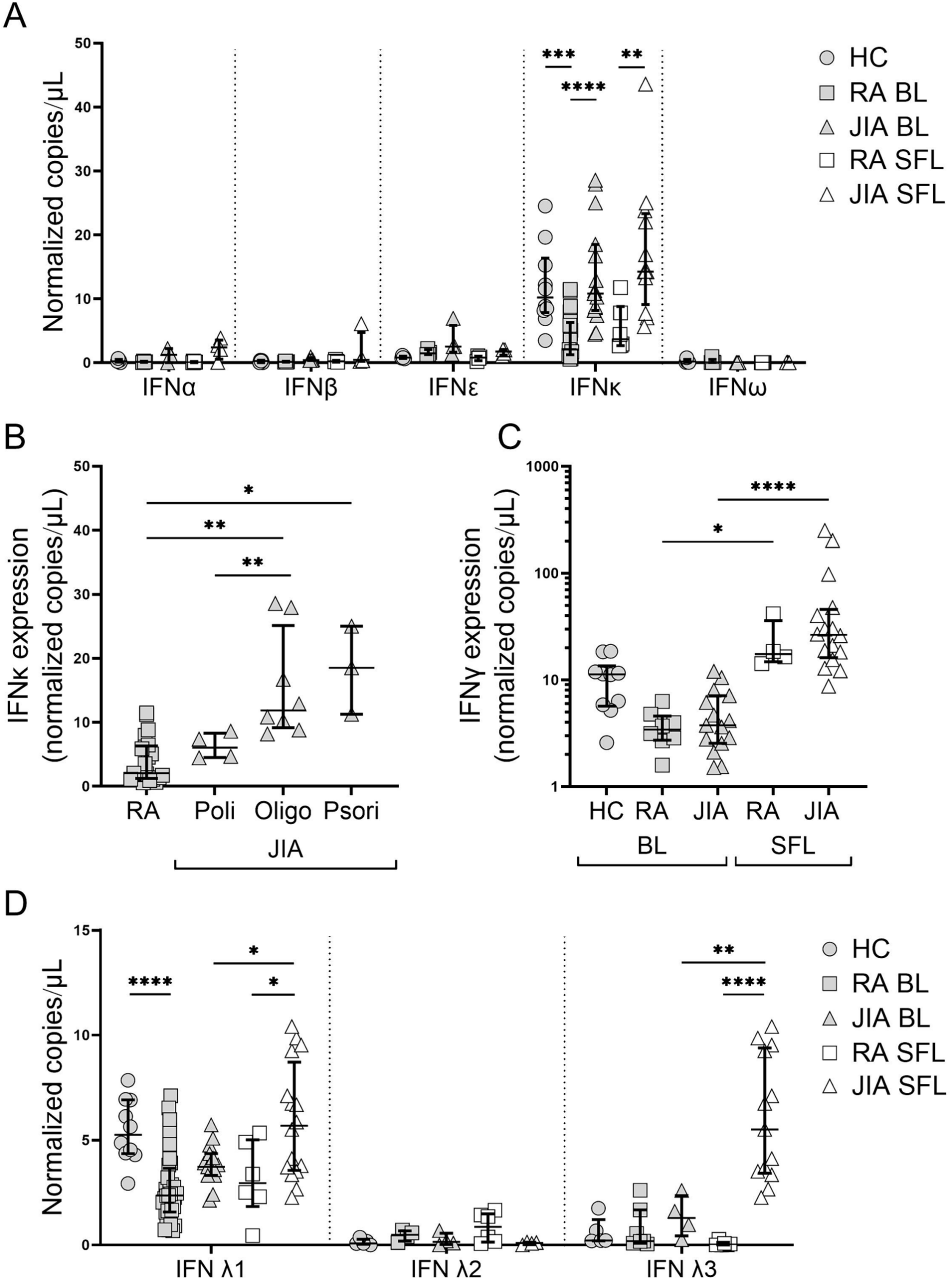
Differential expressions of type I, type II and type III IFNs in JIA and RA. **(A,C)** Type I and type II interferon expression in healthy control (HC), blood leukocytes (BL) and treatment-naïve RA and JIA patients’ BL and synovial fluid leukocytes (SFL). Data were normalized to the expression of reference genes HPRT1 and ACTG1 and show the median and interquartile range of of 29–35 individuals per group for IFNκ and IFNγ, and n = 4–5 for IFNα1, IFNβ1, IFNε and IFNω1. *p < 0.05; **p < 0.01, ***p < 0.001; ****p < 0.0001 by Mann–Whitney test or Wilcoxon test for matched comparisons. **(B)** IFNκ expression among BL in RA and JIA subtypes including polyarticular (Poly), oligoarticular (Oligo) and psoriatic (Psori) JIA. *p < 0.05 by Kruskal-Wallis multiple comparison test with Dunn’s *post hoc* analysis. **(C)** Type II and **(D)** Type III interferon expression in healthy control (HC) blood leukocytes (BL) and treatment-naïve RA and JIA patients’ BL and synovial fluid leukocytes (SFL). Data were normalized to the expression of reference genes HPRT1 and ACTG1 and show the median and interquartile range of 4–18 individuals per group. *p < 0.05; **p < 0.01, ***p < 0.001, ****p < 0.0001 by Mann–Whitney test or Wilcoxon test for matched comparisons.

RNA expression of selected type I (α1, β1, *ε*, κ, ω1), type II (*γ*) and type III (λ1, λ2, λ3) IFNs in HCs and treatment-naïve RA and JIA patients was determined by ddPCR. The expression of IFNα1, IFNβ, IFNε and IFNω1 mRNA was low to undetectable in all BL and SFL patient and HC samples, while IFNκ and IFNγ were readily detectable in all groups (Figure 1). RA BL had significantly lower IFNκ expression compared to HCs and JIA patients (Figure 1A). JIA SFL showed significantly higher IFNκ expression compared RA SFL (Figure 1A). Grouping JIA patients based on disease subtype, revealed higher BL IFNκ expression among oligoarticular and psoriatic JIA patients, compared to RA patients (Figure 1B). The polyarticular JIA samples were similar to that of patients with RA. There was no difference in IFNλ1 and IFNλ3 expression in JIA subtypes.

### Type II IFNγ expression was highly elevated at the site of inflammation

IFNγ was significantly higher in the SFL of RA and JIA patients compared to the matched BL (Figure 1C). No significant differences of BL IFNγ expressions were observed among HCs, RA, and JIA patients, or JIA subtypes (Supplementary Figure 1A).

### Type III IFNλ1 was differentially expressed in RA and JIA

Evaluation of type III IFNs demonstrated that IFNλ1 was significantly lower in RA patients’ BL compared to HCs (Figure 1D). IFNλ2 and IFNλ3 expression in BL was low or undetectable across all groups (Figure 1D). Like IFNκ, significantly higher IFNλ1 and IFNλ3 was observed in JIA patient SFL compared to RA SFL. Moreover, IFNλ1 and IFNλ3 expression was higher in SFL compared to BL in JIA but not in RA patients. IFNλ1 protein was measured in both the plasma and SF of JIA patients, and showed higher protein level of IFNλ1 in the SF (Supplementary Figure 2). No differences in IFNλ1 and IFNλ3 expression were found among JIA subtypes (Supplementary Figures 1B,C).

We found no correlation between levels of IFN expression and baseline DAS28-ESR, inflammatory parameters (CRP, ESR), or ACPA/RF serostatus in RA patients (Supplementary Figure 3). The expression of IFNκ, IFNγ and IFNλ1 was compared based on grouping of RA patients relative to response to MTX treatment. No significant differences in expression between patient groups was observed (data not shown).

### IFN-stimulated gene expression in RA and JIA

**Figure 2:**
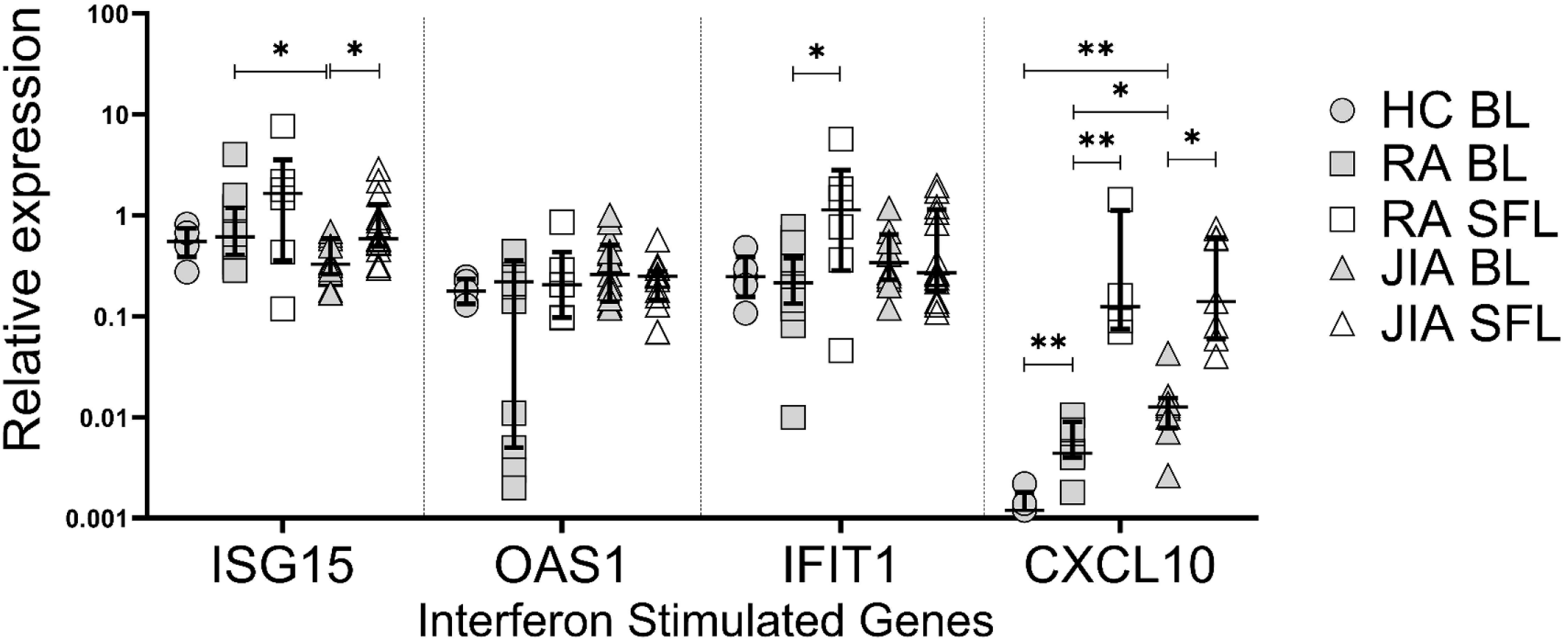
Differential expression of interferon-stimulated genes (ISGs). Relative expression of ISG15, 2′,5′-oligoadenylate synthetase (OAS1), interferon-induced protein with tetratricopeptide repeats 1 (IFIT1), and C-X-C motif chemokine ligand 10 (CXCL10) in healthy control (HC) blood leukocytes (BL) and treatment-naïve RA and JIA patients’ BL and synovial fluid leukocytes (SFL). Data were normalized to the expression level of reference genes HPRT1 and ACTG1 and shown are the median and interquartile range of 5–18 individuals per group. *p < 0.05; **p < 0.01 by Mann–Whitney test or Wilcoxon test for matched comparisons.

The ability of IFN mRNAs expressed in BL and SFLs to stimulate active protein and in turn induce downstream IFN stimulated gene (ISG) message in RA, JIA and control subjects was determined by qPCR in the same samples (Figure 2). We measured ISG15, IFIT1 and OAS1 mRNA expressions to assess interferon signature in our samples. These genes are commonly used as markers to measure the activity of type I and type III interferons because they are strongly upregulated in response to interferon signaling (7). In RA, ISG15, IFIT1 and OAS1 expressions are often elevated, which correlates with disease activity; the overexpression of these genes can lead to increased inflammation and joint damage (24). CXCL10 was used to measure the downstream effect of type II IFNγ. We found, that *ISG15*, *IFIT1* and *CXCL10* mRNA expression were increased in SFL compared to BL. This difference was greatest in the case of *CXCL10* but *IFIT* in RA SFL and *ISG15* in JIA SFL were significantly higher than in BL from the same groups. *CXCL10* mRNA was higher in RA and JIA BL compared to HCs. No differences in SFL ISGs were observed between RA and JIA patients.

ISG expression did not correlate with clinical parameters or disease activity. However, IFNκ correlated with ISG15 (*r* = 0.59 and *p* = 0.03) and IFIT1 (*r* = 0.63 and *p* = 0.02) expression in the BL of JIA patients (Supplementary Figure 4). ISG expression did not differ between RA MTX responders and non-responders (data not shown). IFNγ showed a positive trend with CXCL10 (Supplementary Figure 5).

### Distinct IFN mRNA expression by DC subsets in the synovium

**Figure 3:**
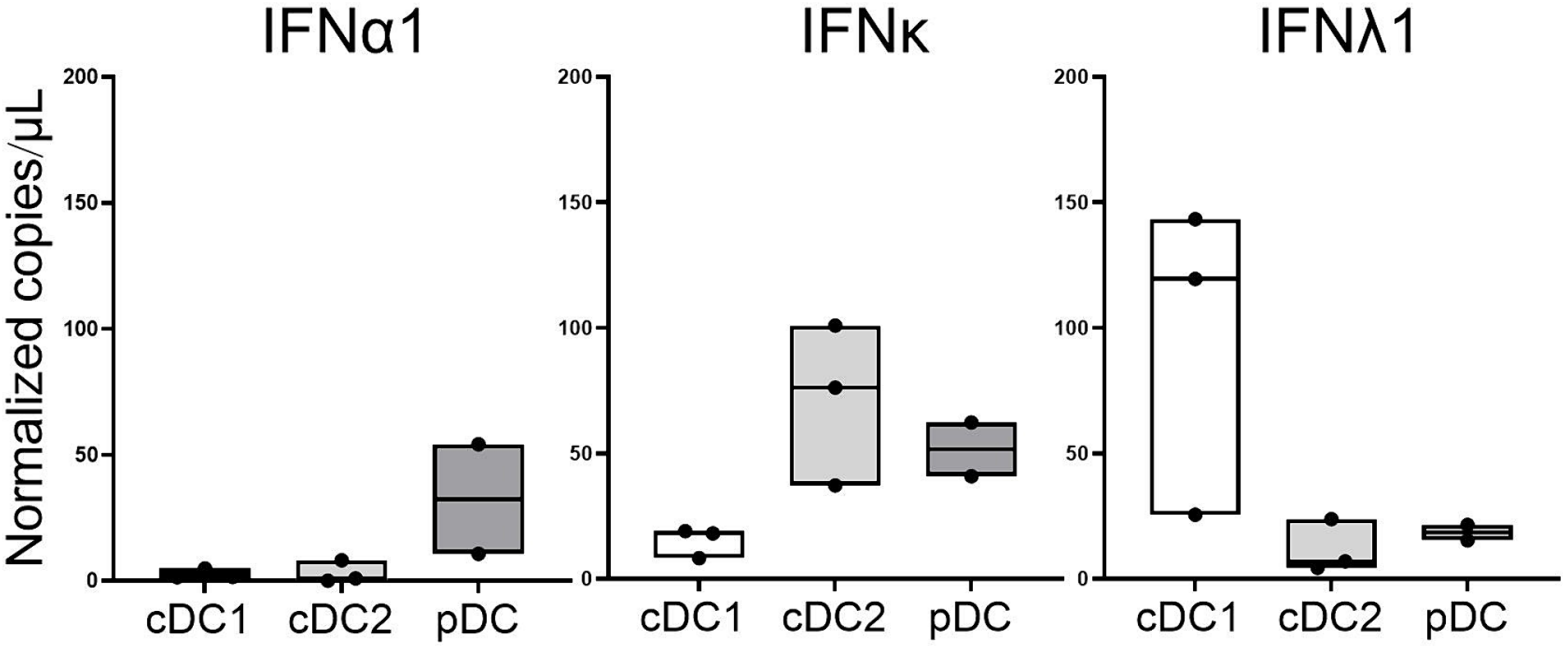
Dendritic cells subsets from JIA synovial fluid express distinct IFN profiles. Dendritic cell subsets (CD11^+^CD141^+^ cDC1, CD11^+^CD1^+^ cDC2, CD11^−^CD123^+^ pDC) were sorted from SFMNC of oligoarticular JIA patients. Expression of selected type I and type III IFNs was measured by ddPCR. Data are the median and the range, symbols represent the individual patient samples (*n* = 3 in case of cDC1 and cDC2; *n* = 2 in case of pDC).

DCs are a major source of type I and type III IFNs (25). To investigate which DC subsets (cDC1: CD11c^+^CD141^+^, cDC2: CD11c^+^CD1c^+^, pDC: CD11c^−^CD123^+^) may be the source of the increased expression of IFNκ and IFNλ1 in the inflamed joint, DCs were sorted from SFMNC of oligoarticular JIA patients (*N* = 3; Supplementary Figure 6). IFNα1 mRNA was primarily produced by pDCs as expected (Figure 3). However, IFNκ was expressed primarily by cDC2 and pDC subsets, and IFNλ1 was mainly expressed by cDC1 cells (Figure 3).

### Type I and type III IFN production by JIA FLS

**Figure 4:**
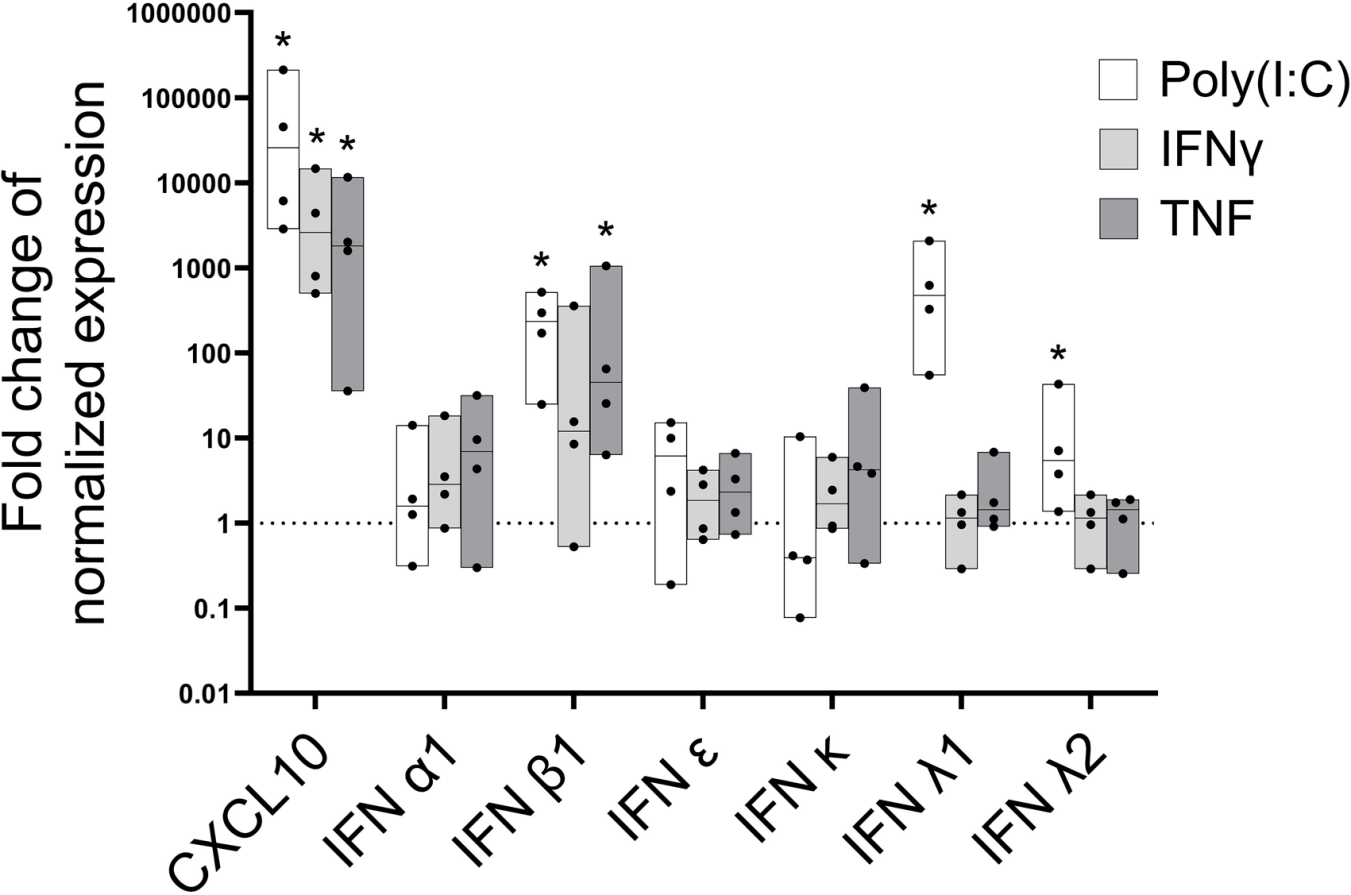
Fibroblast-like synoviocytes contribute to the inflammatory environment by producing specific type I and type III interferons in JIA patients. Fibroblast-like synoviocytes (FLS) were treated with poly(I:C; 10 mg/mL), IFNγ (100 IU/mL), or tumor necrosis factor (TNF; 50 ng/mL) for 6 h. Type I IFNs (IFNα1, IFNβ, IFNε, and IFNκ) and type III IFNs (IFNλ1 and IFNλ2) mRNA induction were measured by qPCR. The interferon-stimulated gene CXCL10 was a positive control. Data are shown as the fold change relative to unstimulated control of normalized expression values (using reference genes HPRT1 and ACTG1). Data show the median, maximum, and minimum values of cultures from four patients. *p < 0.05 by Wilcoxon-test. The dotted line represents the normalized expression in non-treatment controls.

Although DCs are the primary source of type I and III IFNs, other cells can also produce these cytokines (26). Primary JIA FLS were stimulated with TNF, IFNγ and the TLR3 ligand poly(I:C). The increase in expression of IFNs and (the positive control) CXCL10 was determined relative to unstimulated FLS, in which IFN expression was very low or undetectable (Figure 4). IFNβ expression was significantly induced by poly(I:C) and TNF, while IFNλ1 and IFNλ2 expression were induced exclusively by poly(I:C) and not the cytokines (Figure 4). CXCL10 was strongly induced by all stimuli.

## Discussion

Our study identified striking differences in the expression of type I and type III IFNs in RA and JIA. Of the numerous type I IFNs, IFNκ was the only type I IFN detected in substantial amounts. RA BL and SFL expressed very little IFNκ, with BL expression significantly less than that in HC. JIA BLs and SFLs expressed IFNκ, and this was significantly more than that in RA leukocytes. IFNγ was detectable in both RA and JIA and it was significantly higher in SFL than BL. Type III IFNs, like type I IFNs, were also low in RA leukocytes, while elevated expressions of IFNλ1 and IFNλ3 were readily detectable in JIA SFL and significantly higher than in RA SFLs. IFNλ3 was much higher in JIA SFL than in RA, where none was present. Interestingly, despite these differences in IFN expression, some ISG expression was elevated in both RA and JIA SFL. This also demonstrated that the IFN mRNA induced IFN proteins and, in turn, activated ISGs. Interestingly, CXCL10 was the only ISG which consistently showed an increased expression in BL of arthritic subjects compared with healthy controls.

Our novel finding of IFNκ expression in JIA, particularly in oligoarticular and psoriatic subtypes, compared to polyarticular JIA and RA, suggests a role for IFNκ in JIA pathogenesis. IFNκ plays a nuanced role in human diseases (27). Unlike IFNα and IFNβ, IFNκ has unique characteristics and expression patterns. In the skin, IFNκ is predominantly expressed in keratinocytes (28). It is elevated in keratinocytes following viral infection, exposure to double-stranded RNA, or IFNγ and IFNβ (29). It was shown, that serum IFNκ was elevated in inflamed skin diseases, such as cutaneous lupus and psoriasis (30, 31). IFNκ is also produced by monocytes and DCs (32). The finding that IFNκ expressions are low in both polyarticular JIA and RA aligns with the concept of shared characteristics between these conditions, potentially informing JIA classification and may be useful in management strategies (2, 33). Understanding the mechanisms behind the differential IFNκ expression in specific JIA subtypes may reveal distinct disease-specific pathways and offer new therapeutic options.

IFNα, IFNβ, IFNε and IFNω1 mRNA were low to undetectable in BL and SFLs from both RA and JIA patients. Previous studies suggest that soluble factors, such as TGFβ, may impair the ability of pDCs to mature and release type I IFNs into RA SF (34). These regulatory factors may account for the low or absent levels measured in our patients. Our results suggest distinct pathways regulating IFNκ. Others have reported better responses to TNF-inhibitors in RA patients with a lower IFNβ to IFNα ratio (35, 36). However, in these studies, the IFN activity was measured by reporter cell assays using patient’s serum in the presence of anti-IFNα or anti-IFNβ, and the activity of other type I and type III IFNs and IFN message in blood leukocytes were not determined.

Prior information on the expression of type III IFNs in JIA is limited to a single study reporting lower intraocular IFNλ1 in JIA-associated uveitis compared to idiopathic uveitis (36). We found elevated IFNλ1 and IFNλ3 expression in SFL at the site of inflammation but not in the blood. This is consistent with data showing high IFNλ in the synovial tissue of adult patients with autoimmune rheumatic diseases (37). We also demonstrated that IFNλ1 is primarily produced by the CD141^+^ cDC1s, which are likely one of the key regulators of the immune response in the joint, such as found in RA and psoriatic arthritis (38).

To our knowledge, we are the first to measure IFNλ2 expression in JIA SFL. We found that IFNλ2 expression was low to undetectable in BL and SFL. In a mouse model of RA, IFNλ2 treatment reversed arthritis by suppressing IL-1β and restricting neutrophil recruitment to inflammatory sites (39). The lack of IFNλ2 in both RA and JIA suggests a possible loss of these anti-inflammatory effects in arthritis. Insufficient IFNλ2 levels may shift the balance toward a pro-inflammatory state. Hypothetically, therapeutic administration of recombinant IFNλ2 could help resolve arthritis in patients by restoring anti-inflammatory functions; this may represent a novel biological treatment approach to consider. The low levels of endogenous IFNλ2 suggests that specifically blocking IFNλ1 may be a more beneficial strategy than generalized IFNλ inhibition.

Preliminary investigations into the role of specific DC subsets revealed that cDC2 cells are a significant source of IFNκ production. In contrast, cDC1 cells predominantly expressed IFNλ1, underscoring the potential subtype-specific roles of DCs in modulating IFN responses. The purpose of this analysis was to identify which DC subsets were responsible for producing specific IFNs within the synovial fluid. The DCs were isolated from the synovial fluid of three patients, who had oligoarticular JIA. While the JIA subtype could influence the overall level of IFN expression, we believe that it should not affect the cellular source of the IFNs, which was the focus of this experiment.

In addition, the TLR3 agonist poly(I:C) upregulated not only IFNβ, but IFNλ1 and IFNλ2 expression in the FLS from JIA patients suggesting that FLS can also produce both type I and type III IFNs and likely contribute to the inflammatory environment in the joint. Synoviocytes are the dominant non-immune cells of the synovial tissue and contribute to joint destruction via multiple mechanisms (40). These findings suggest which synovial cells could be therapeutic targets, although macrophages, T and B cells may also contribute substantially to IFN production. Understanding IFN sources in the joint facilitates selectively targeted delivery of pathway inhibitors to relevant cell populations.

The IFN pathway is an attractive therapeutic target, and several drugs are currently under investigation for treatment of systemic autoimmune diseases (41). Biologics blocking the IFN receptor subunits of IFNAR1 or IFNLR1 may have differential effects based on IFN profiles. The distinct IFN profiles among arthritis subtypes imply that targeted anti-IFN treatments need to be tailored based on disease specifics. The varying abundance of specific IFNs between disease subtypes suggests relative efficacy of JAK inhibitors may differ. For example, higher synovial IFNλ1 in JIA indicates JAK inhibitors may have greater benefit in JIA by inhibiting IFNλ1 activity, versus RA where IFNλ1 is lower. IFNκ inhibitors may have particular utility in oligo/psoriatic JIA given the high levels observed. Additionally, patients with very high synovial IFN levels may benefit most from localized intra-articular anti-IFN agents. Measuring IFN subtype levels could help identify patients most likely to respond to JAK pathway blockade based on their dominant IFN species. Our findings support the development of more selective inhibitors of specific IFN subtypes, as opposed to broad JAK inhibitors (42). Moreover, the reduced IFNλ2 levels imply loss of natural inflammation control mechanisms in arthritis pathogenesis. Boosting IFNλ2 could theoretically exert protective actions, while IFNλ2-sparing interventions may be preferred therapeutically to maintain any residual anti-inflammatory activity. Further mechanistic studies on the drivers of diminished IFNλ2 in arthritis are warranted.

The small sample size is a limitation of this study, underscoring the need for larger, more comprehensive investigations. In addition, longitudinal studies with objective clinical parameters would evaluate the predictive role of type I and type III IFNs on the disease outcome in JIA patients.

In conclusion, our findings suggest that IFNκ and the type III IFNs, plays a more prominent role in the pathophysiology of JIA compared to RA, especially in oligoarticular and psoriatic JIA subtypes. This supports the concept that the pathophysiology of these conditions is influenced by unique IFN-mediated pathways, underscoring the need for subtype-specific approaches in their management and potentially more targeted and effective therapeutic strategies.

## Data Availability

The original contributions presented in the study are included in the article/Supplementary material, further inquiries can be directed to the corresponding author.
