# Supplementary material for: Differential type I and type III interferon expression profiles in rheumatoid and juvenile idiopathic arthritis
Source: Front Med (Lausanne). 2024 Sep 27;11:1466397. doi: 10.3389/fmed.2024.1466397 (PMC11468860; doi:10.3389/fmed.2024.1466397)
Supplement: Supplementary file 1 [file Data_Sheet_1.PDF]

Supplementary Table 1. Antibodies and reagents used for immunofluorescent staining

Marker	Clone	Fluorochrome	Supplier
CD1c	F10/21A3	BB515	BD Biosciences
CD3	SK7	BV786	BD Biosciences
CD11c	B-ly6	BV650	BD Biosciences
CD14	M5E2	BV605	BD Biosciences
CD19	HIB19	BUV496	BD Biosciences
CD56	5.1H11	BV570	BioLegend
CD123	7G3	PE-Cy7	BD Biosciences
CD141	1A4	BV711	BD Biosciences
Viability Dye	-	FVS575V	BD Biosciences
