# Supplementary material for: Differential type I and type III interferon expression profiles in rheumatoid and juvenile idiopathic arthritis
Source: Front Med (Lausanne). 2024 Sep 27;11:1466397. doi: 10.3389/fmed.2024.1466397 (PMC11468860; doi:10.3389/fmed.2024.1466397)
Supplement: Supplementary file 2 [file Data_Sheet_2.PDF]

Supplementary Table 2. Target information of qPCR and ddPCR primers

	Gene symbol	Sequence accession number	Manufacturer	Assay ID	Amplicon length	Amplicon sequence/Reference position
Reference genes	***ACTG1***	NC_000017.10, NG_011433.1, NT_010783.15	Bio-Rad	qHsaCED0005010	191	AGATACAAGCTTCAAGGACAATTTCTTTTCGAAGGCTTATTCCAGTTTCGTGAGG CTAGCATGAGGTGTGTGCATTTGCCAGGGGCAAATTTCTATTCTCAATTAACCCA TGCAGCAAATGCTACGCATCTGCTGAGTCCGTTTAGAAGCATTTGCGGTGGACG ATGGAGGGGCCCGACTCGTCGTACTCCTGCTTGCTAATCCACATCTGCTGGAAG GTG
	HPRT	NC_000023.10, NG_012329.1, NT_011786.16	Bio-Rad	qHsaCID0016375	90	GACACTGGCAAAACAATGCAGACTTTGCTTTCCTTGGTCAGGCAGTATAATCCAA AGATGGTCAAGGTCGCAAGCTTGCTGGTGAAAAGGACCCCACGAAGTGTTGGAT ATAAGCCAGAC
Type I interferons	***IFN α 1***	NM_024013	Qiagen	PPH01321B-200	60	709
	IFN β 1	NM_002176	Qiagen	PPH00384F-200	148	332
	IFN ε	NM_176891	Qiagen	PPH21033A-200	98	655
	IFN κ	NM_020124	Qiagen	PPH15948E-200	119	325
	IFN ω 1	NM_002177	Qiagen	PPH01072A-200	119	1087
Type II interferon	***IFN γ***	NC_000012.11, NG_015840.1, NT_029419.12	Bio-Rad	qHsaCED0043378	98	CACAACCCATGGGATCTTGCTTAGGTTGGCTGCCTAGTTGGCCCCTGAGATAAA GCCTTGTAATCACATAGCCTTGCCTAATTAGTCAGAAAACAAAGGATTAAGTGAG ACAGTCACAGGATATAGGA
Type III interferons	***IFN λ 1***	NC_000019.9, NT_011109.16	Bio-Rad	qHsaCED0003353	85	GAGTCCGCTGGCTGCCTGGAGGCATCTGTCACCTTCAACCTCTTCCGCCTCCTC ACGCGAGACCTCAAATATGTGGCCGATGGGAACCTGTGTCTGAGAACGTCAACC CACCCTG
	IFNλ2	NC_000019.9, NT_011109.16	Bio-Rad	qHsaCED0057428	118	CACCTTCAACCTCTTCCGCCTCCTCACGCGAGACCTGAATTGTGTTGCCAGTGG GGACCTGTGTGTCTGACCCTCCCACCAGTCATGCAACCTGAGATTTTATTTATAA ATTAGCCACTTGTCTTAATTTATTGCCACCCAGTCGCTA
	IFNλ3	NC_000019.9, NT_011109.16	Bio-Rad	qHsaCED0038284	94	CGCGGAGCCTGGCGACAGGAACTGCTCCAGTCACGGTCAGCACTGCGGCCATC AGCACCAGCACTGGCATGCAGTCCCCGGTCATGTCTGTGTCACAGAGAGAAAG GGAGCTGAGGGAATGCAG
Interferon stimulated genes	***CXCL10***	NM_001565	Qiagen	PPH00765E-200	111	800
	IFIT1	NM_001548	Qiagen	PPH01332F-200	93	1544
	ISG15	NM_005101	Qiagen	PPH01333F-200	154	355
	OAS1	NM_002534	Qiagen	PPH01324A-200	112	1076

*ACTG1, actin gamma 1; HPRT, Hypoxanthine Phosphoribosyl transferase 1; IFN, interferon; CXCL10, C-X-C motif chemokine ligand 10; IFIT1, Interferon Induced Protein with Tetratricopeptide Repeats 1; ISG15, Interferon-stimulated gene product 15; OAS1, 2'-5'-Oligoadenylate Synthetase 1
