# Supplementary material for: Differential type I and type III interferon expression profiles in rheumatoid and juvenile idiopathic arthritis
Source: Front Med (Lausanne). 2024 Sep 27;11:1466397. doi: 10.3389/fmed.2024.1466397 (PMC11468860; doi:10.3389/fmed.2024.1466397)
Supplement: Supplementary file 3 [file Data_Sheet_3.PDF]

Supplementary Table 3. Characteristics of the enrolled research subjects at baseline.

	HC	RA total	RA MTX responders	RA MTX non-responders	JIA
	(n = 10)	(n=35)	(n = 15)	(n = 20)	(n= 16)
Age, mean ± SD years	45.3 ± 11.5	63.7 ± 11.5	63.9 ± 12.7	63.5 ± 10.8	6.23 ± 4.78
Sex, no. female/male	6/4	21/14	9/6	12/8	9/7
CRP, median (IQR) mg/liter	–	10.5 (4.8-21.6)	7.9 (3.7-12)	15.4 (6.8-23.3)	–
ESR, median (IQR) mm/hour	–	29 (14-42)	21.0 (12-31)	36.5 (16-49.5)	–
DAS28-ESR, median (IQR)	–	5.4 (5.0-6.4)	5.4 (4.9-5.9)	5.8 (5.1-6.6)	–
ACPA, number of positive/negative	–	21/14	9/6	12/8	–
RF, number of positive/negative	–	22/13	9/6	13/7	–
ANA, number of positive/negative	–	–	–	–	10/6
JIA subtypes, number of oligo/poly/psoriatic	–	–	–	–	9/4/3
Symptom duration, median (IQR) months	–	6.5 (4-19)	7.0 (3-21)	5.5 (4-15)	6.5 (2-21)

HC = healthy controls; RA = rheumatoid arthritis; MTX = methotrexate; JIA = juvenile idiopathic arthritis; CRP = C-reactive protein; IQR = interquartile range; ESR = erythrocyte sedimentation rate; DAS28-ESR = Disease Activity Score in 28 joints using the ESR; ACPA = anti-citrullinated protein antibody; RF = rheumatoid factor; ANA = antinuclear antibody; oligo = oligoarticular; poly = polyarticular.
