# Supplementary material for: Differential type I and type III interferon expression profiles in rheumatoid and juvenile idiopathic arthritis
Source: Front Med (Lausanne). 2024 Sep 27;11:1466397. doi: 10.3389/fmed.2024.1466397 (PMC11468860; doi:10.3389/fmed.2024.1466397)
Supplement: Supplementary file 4 [file Data_Sheet_4.PDF]

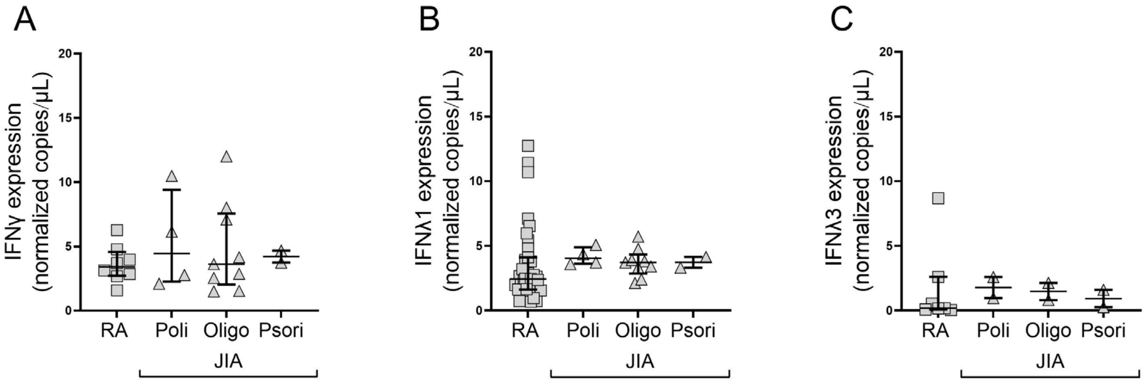

Supplementary Figure 1. Type II and type III IFN expression in RA and JIA subtypes. IFNκ expression among BL in RA and JIA subtypes including polyarticular (Poly), oligoarticular (Oligo) and psoriatic (Psori) JIA. *=P < 0.05 by Kruskal-Wallis multiple comparison test with Dunn's post hoc analysis.
