# Supplementary material for: Differential type I and type III interferon expression profiles in rheumatoid and juvenile idiopathic arthritis
Source: Front Med (Lausanne). 2024 Sep 27;11:1466397. doi: 10.3389/fmed.2024.1466397 (PMC11468860; doi:10.3389/fmed.2024.1466397)
Supplement: Supplementary file 5 [file Data_Sheet_5.PDF]

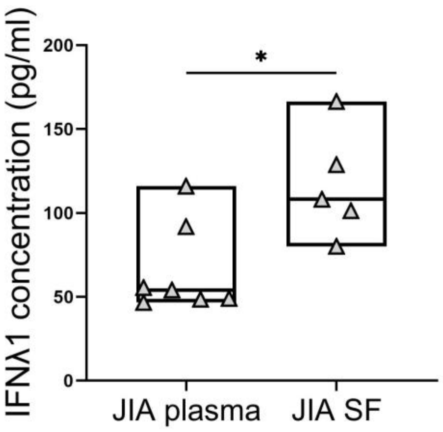


**Supplementary Figure 2. IFNλ1 protein level in the plasma and synovial fluid (SF) of JIA patients.** Data are the median and range, the symbols represent individual samples. *=P < 0.05 by Mann-Whitney test.
