# Supplementary material for: Differential type I and type III interferon expression profiles in rheumatoid and juvenile idiopathic arthritis
Source: Front Med (Lausanne). 2024 Sep 27;11:1466397. doi: 10.3389/fmed.2024.1466397 (PMC11468860; doi:10.3389/fmed.2024.1466397)
Supplement: Supplementary file 6 [file Data_Sheet_6.PDF]

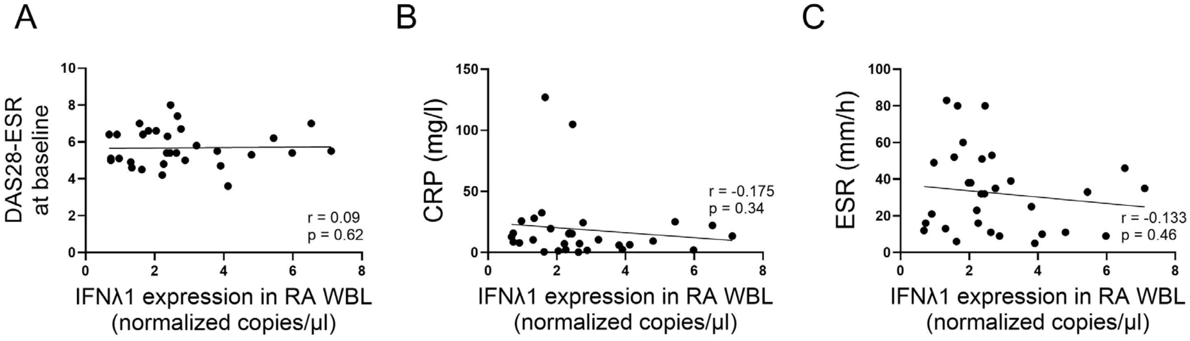

Supplementary Figure 3. IFNλ1 mRNA expression in whole blood leukocytes (WBL) did not correlate with clinical parameters in RA patients.

The symbols represent individual samples. Correlation calculated by Spearman method.
