# Supplementary material for: Differential type I and type III interferon expression profiles in rheumatoid and juvenile idiopathic arthritis
Source: Front Med (Lausanne). 2024 Sep 27;11:1466397. doi: 10.3389/fmed.2024.1466397 (PMC11468860; doi:10.3389/fmed.2024.1466397)
Supplement: Supplementary file 7 [file Data_Sheet_7.PDF]

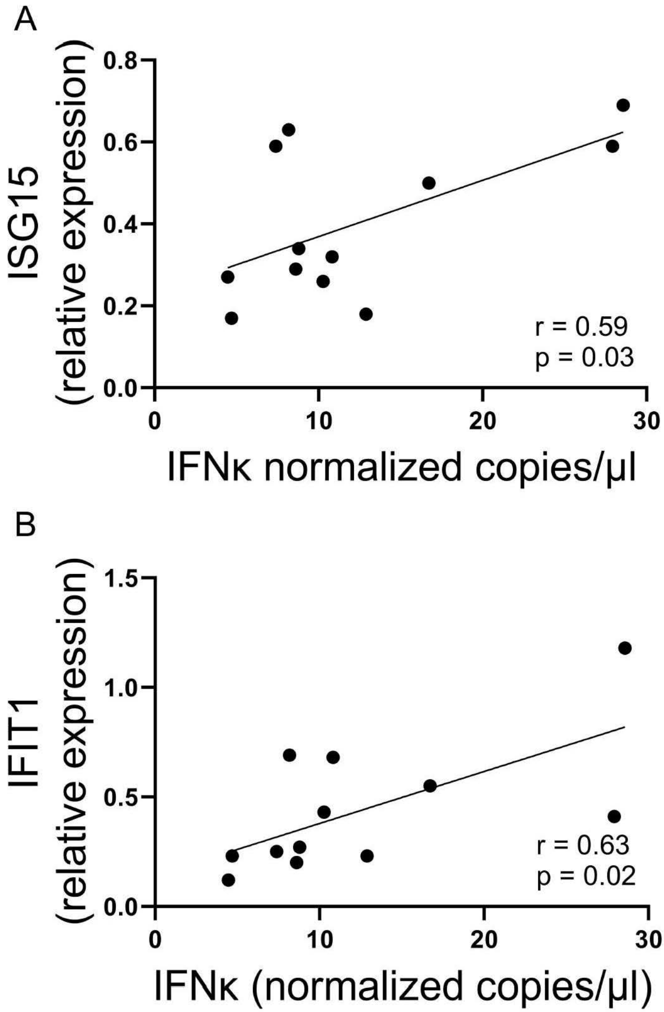

Supplementary Figure 4. IFNκ expression correlates with ISG15 and IFIT1 IFN-stimulated genes (ISG) in the blood leukocytes of JIA patients. **A** and **B**, ISG15 and IFIT1 expression was measured by qPCR, IFNκ expression was measured by ddPCR in the blood leukocytes in JIA patients. Symbols represent individual values. N=12. Spearman's rank correlation coefficient (r) and corresponding p-value are shown. $p < 0.05$ and $r = 0.5$ to 0.7 indicating a moderate positive correlation between IFNκ and these ISGs.
