# Supplementary material for: Differential type I and type III interferon expression profiles in rheumatoid and juvenile idiopathic arthritis
Source: Front Med (Lausanne). 2024 Sep 27;11:1466397. doi: 10.3389/fmed.2024.1466397 (PMC11468860; doi:10.3389/fmed.2024.1466397)
Supplement: Supplementary file 8 [file Data_Sheet_8.PDF]

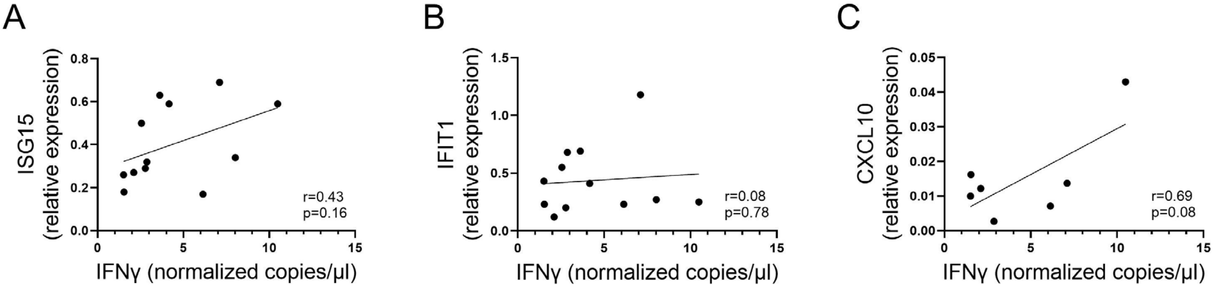

Supplementary Figure 5. IFNγ mRNA expression in whole blood leukocytes (WBL) showed a positive trend with CXCL10 mRNA expression.

The symbols represent individual samples. Correlation calculated by Spearman method.
