# Supplementary material for: Differential type I and type III interferon expression profiles in rheumatoid and juvenile idiopathic arthritis
Source: Front Med (Lausanne). 2024 Sep 27;11:1466397. doi: 10.3389/fmed.2024.1466397 (PMC11468860; doi:10.3389/fmed.2024.1466397)
Supplement: Supplementary file 9 [file Data_Sheet_9.PDF]

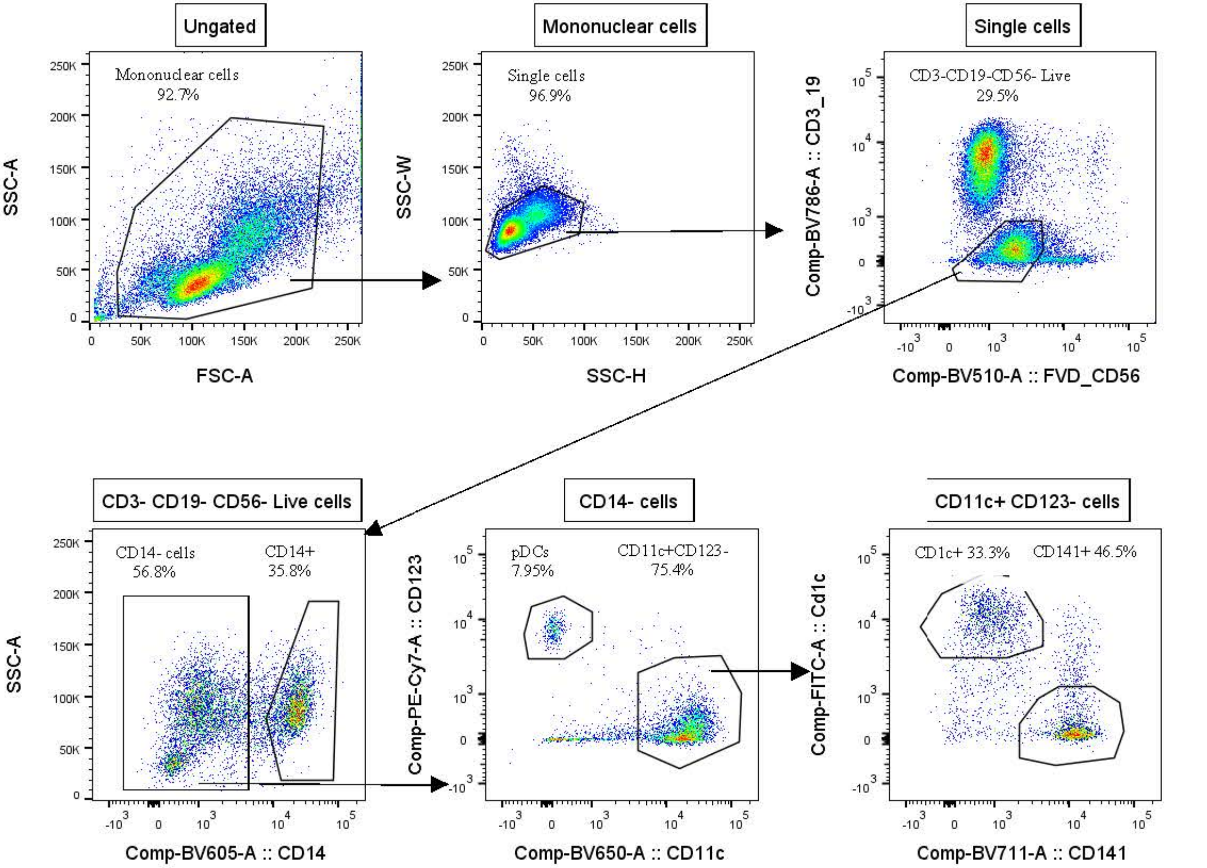

Supplementary Figure 6: Dendritic Cell Subset Sorting Strategy from JIA Patient Synovial Fluid Mononuclear Cells. Initially, single cells were identified and gated. Lymphocytes (CD3+), B cells (CD19+), and NK cells (CD56+) were excluded from the population. Subsequently, monocytes (CD14+) were also removed from the analysis. The gating strategy then focused on identifying plasmacytoid dendritic cells (pDCs) via positive CD123 expression. CD11c+ myeloid cells that were negative for CD123 (CD123-) were further differentiated. Within this population, two distinct dendritic cell subsets were identified and sorted: CD141+ cells corresponding to conventional dendritic cells type 1 (cDC1) and CD1c+ cells corresponding to conventional dendritic cells type 2 (cDC2).
